# Exploring local adjuvant antibiotics as viable alternative to systemic antibiotics in non-surgical periodontal therapy: Clinical, immunological and microbiological insights

**DOI:** 10.1371/journal.pone.0333342

**Published:** 2025-09-29

**Authors:** Iva Milinkovic, Marija Vuckovic, Nadja Nikolic, Jelena Carkic, Zoran Aleksic, Sasha Jankovic, Jelena Milasin, Ana Djinic Krasavcevic

**Affiliations:** 1 Department of Periodontology and Oral Medicine, School of Dental Medicine, University of Belgrade, Belgrade, Serbia; 2 Implant-Research Centre, School of Dental Medicine, University of Belgrade, Belgrade, Serbia; Danube Private University, AUSTRIA

## Abstract

**Objectives:**

The primary aim of the present study was to compare the reduction in periodontal probing depth after the use of either local or systemic adjuvant antibiotics during non-surgical periodontal therapy at baseline and the six-month follow-up. Secondary aims were to compare other clinical outcomes, total bacterial count, and relative expression levels of certain pro-inflammatory mediators following the use of locally and systemically delivered adjuvant antibiotics during the initial non-surgical periodontal therapy (NSPT).

**Material and methods:**

A total of 38 periodontitis stage III grade B or C patients were randomly and equally assigned to receive either local (LA) (tazobactam + piperacillin preparation) or systemic antibiotics (SA) (amoxicillin and metronidazole combination) during NSPT. Clinical periodontal parameters (periodontal probing depth, clinical attachment level, bleeding on probing, and plaque index), and microbiological and immunological parameters: total bacterial count, and relative expression levels of IL-17 and TNF-α were evaluated at baseline and six months after NSPT.

**Results:**

Both LA and SA significantly improved all clinical parameters after six months (*p*<0.001 for all comparisons). While a significant decrease of total bacterial count was observed after LA (*p*=0.001) and SA treatment (*p*=0.013), the insignificant differences between the two groups were observed after the sixth-month follow-up (*p*>0.05). Conversely, relative gene expression levels of IL-17 and TNF-α did not differ significantly between LA and SA six months after the NSPT. The insignificant differences between these cytokines’ relative levels were observed in both SA and LA between baseline and follow-up measurements.

**Conclusions:**

The adjuvant use of piperacillin + tazobactam led to the comparable improvement of clinical, microbiological and immunological parameters to the conventional use of amoxicillin and metronidazol combination six months following the initial therapy of periodontitis. Therefore, these locally delivered antibiotics might be a promising alternative to the standard use of systemic amoxicillin and metronidazol combination during initial therapy of stage III periodontitis.

## 1. Introduction

Periodontitis is a chronic, multifactorial inflammatory disease resulting in periodontal attachment loss and tooth‐supporting tissues’ progressive destruction. It is characterised by microbially-associated and host‐mediated inflammation [1,2]. The disease onset and progression hinge on abundant biofilm accumulation, dysbiotic ecological changes in the microbiome, and an aberrant host immune response [2,3]. As a consequence of an inadequate immune response to periodontal pathogens, an excessive accumulation of immunomodulatory cells occurs, leading to the breakdown of tissue homeostasis and subsequent alveolar bone loss [3].

The current literature provides numerous data on the roles of various pro-inflammatory mediators in the mechanisms of periodontitis progression. Tumor necrosis factor-alpha (TNF-α) is considered a key cytokine playing a critical role in the innate response against the periodontal pathogens [4]. TNF-α appears to be involved in the induction of other inflammatory mediators, endothelial cell activation, and endothelial-leukocyte interactions [5], as well as in bone remodelling processes where it contributes to bone resorption [6]. Interleukin-17 (IL-17) has a prominent role in immune surveillance at mucosal and barrier surfaces; however, it has also been increasingly implicated as a driver of immunopathology in settings of autoimmunity and chronic inflammation [7]. Additionally, IL-17 takes part in osteolysis mechanisms through an excessive neutrophil recruitment, enhancing other pro-inflammatory cytokine production, and activating osteoclasts [8].

Non-surgical periodontal therapy (NSPT) is the initial treatment phase, a crucial and integral therapy part. The goal of NSPT is the removal of all bacterial deposits and the reduction of inflammation by means of mechanical debridement. The reduction of bacterial count below individual threshold levels of inflammation/disease establishes an adequate infection control [9]. Although NSPT alone leads to significant improvement of the disease, the adjunctive use of antibiotics during NSPT has been significantly favourable in terms of clinical outcomes [10–12]. Particularly the significant improvement of periodontal probing depth was observed at six-month follow-up [10].

Nevertheless, the development of bacterial resistance due to the overuse of antibiotics has raised global concerns nowadays, leading to very strict recommendations for prescribing systemic antibiotics [13]. Importantly, systemic antibiotic regimens have shown long-lasting impact on the faecal microbiome, including an increase in genes associated with antimicrobial resistance. Therefore, due to the serious concerns of the impact of systemic antibiotics on both individual patients’ health and public health, their routine use as an adjunct to NSPT is not recommended [13]. Following the latest recommendations, the systemic use of antibiotics alongside NSPT is justified and especially beneficial in younger individuals [14] with generalised periodontitis stage III [13]. In addition, especially patients suffering from generalised stage III, grade C periodontitis obtained clinically relevant greater benefits from systemic amoxicillin and metronidazole combination adjunctive to NSPT than placebo [15].

Particularly, the beneficial changes after NSPT in the subgingival microbial composition, accompanied by important and sustained clinical improvements, were achieved with the adjuvant use of metronidazole and amoxicillin combination [16]. An *in vitro* study pointed out that metronidazole was able to inhibit the production of TNF-α, among other cytokines [17]. However, comparable observations were not confirmed in clinical settings [18].

In addition to the use of systemic antibiotics, the adjunctive use of locally delivered subgingival antimicrobials may also be considered in periodontitis patients during NSPT [13]. The advantage would be local action and controlled application, that could prevent developing the antimicrobial resistance to medications [19]. In that sense, previous studies also reported the statistically significant benefits regarding periodontal probing reduction and clinical attachment level gain when performing NSPT with locally delivered antimicrobials [20,21]. Conversely, other authors emphasised that the overall outcomes of local antimicrobial treatments were not found to be particularly promising [16], limiting the use of local antimicrobial therapy during the maintenance phase only, for treating remaining and isolated active pockets [22]. Nonetheless, the development of various local antimicrobials preparations has been occurred, providing with potentially useful LA for previously mentioned purpose [23].

Bearing in mind the somewhat controversial data found in present-day literature, the null hypothesis of the present study was set, stating that there was no statistically significant difference in periodontal probing depth between patients receiving systemic antibiotics and those receiving local antibiotics as an adjunct to NSPT. Accordingly, the primary aim of the present study was to compare the reduction of periodontal probing depth after the use of local and systemic adjuvant antibiotics during NSPT, at baseline and six months following the treatment. Furthermore, the secondary aims were differentiating the other clinical outcomes, along with total bacterial count and relative expression levels of certain pro-inflammatory mediators following the use of locally and systemically delivered adjuvant antibiotics during NSPT.

## 2. Materials and methods

### 2.1. Participants, sample size calculation, study design, and data collection

The present single-blinded randomised clinical study included a total of 38 individuals. All participants were recruited at the Department of Periodontology and Oral Medicine, School of Dental Medicine, University of Belgrade, from 17/01/2023–11/03/2024. The laboratory procedures were conducted in the Laboratory for Molecular Biology, Implant-Research Centre, School of Dental Medicine, University of Belgrade. The research was approved by the Ethical Committee of the School of Dental Medicine prior to study initiation (written consent No 36/6). The research was conducted in full accordance with the 2013 revision of the Helsinki Declaration of 1975. All patients signed the written informed consent before inclusion in the study. The informed consent was previously waived by the Ethical Committee of the School of Dental Medicine. Soon after the research approval was obtained from the institutional ethical committee in March 2020, the COVID-19 pandemic began; therefore, the patient recruitment did not start until 2023. Since the European Federation of Periodontology guideline for periodontitis treatment was published in the meantime [11], its restrictive recommendations on systemic adjunct antibiotics use during NSPT were adopted. This study was prospectively registered at ClinicalTrials.gov (NCT 05608564) on 31/10/2022, prior to the commencement of participants’ recruitment, in accordance with ICMJE and CONSORT guidelines. The authors confirm that all ongoing and related trials for this drug/intervention are registered.

The inclusion criteria were:

- Individuals 18–40 years old;
  - Active periodontitis stage III according to the latest criteria, grade B or C [1];
  - Non-smokers and light smokers (up to 10 cigarettes/day).

The exclusion criteria were:

- Individuals younger than 18 years of age (minors);
- Presence of systemic diseases affecting the tooth-supporting apparatus and bone metabolism (uncontrolled diabetes mellitus, cancer, immunodeficiency, metabolic bone diseases);
  - Patients receiving an immunosuppressive, anti-resorptive or anti-inflammatory therapy;
  - Allergy to penicillin;
  - Periodontal therapy within the last 6 months;
  - Use of local and/or systemic antimicrobials within the last 6 months;
  - Use of oral anti-plaque mouthwash at least one month prior to the study;
  - Alcohol or drug abuse;
  - Pregnancy or lactation.

The sample size calculation was based on the requirements of the first phase study and used data from an earlier study [24]. This determined that 17 subjects per treatment arm would provide 80% power to detect a true difference of 1.0 mm between test and placebo using PPD reduction in pockets ≥7 mm as the primary outcome variable, assuming that the common standard deviation is 1.0 mm. Accordingly, a sample of 17 subjects per arm was considered sufficient for our study, also bearing in mind the another relevant study [25].

**Fig 1:**
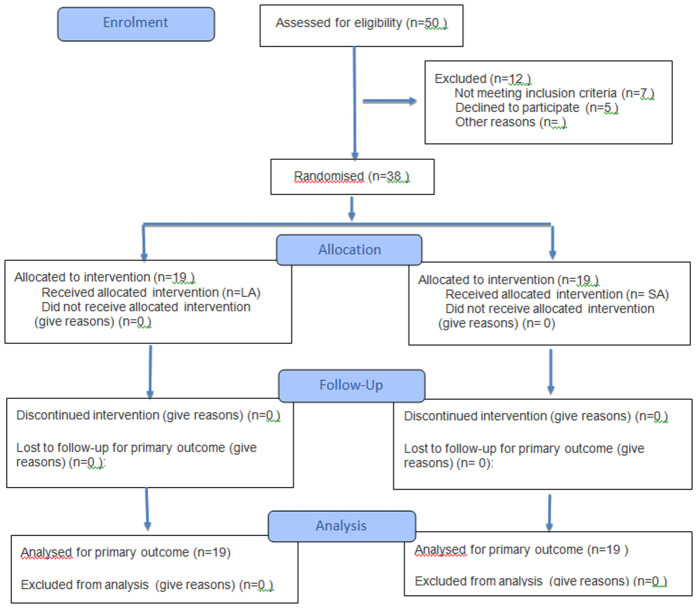
The flow-diagram for the randomised clinical study.

The patients were assessed for eligibility in the study. Following the initial evaluation of 50 individuals, seven patients were excluded due to not fulfilling the study inclusion criteria, and five patients declined to participate in the trial. Finally, 38 participants were allocated in the intervention arm. There were no dropouts during the study protocol (Fig 1).

Clinical periodontal parameters such as periodontal probing depth (PPD), clinical attachment level (CAL), bleeding on probing (BOP) [26], and plaque index (PI) [27] were recorded for each patient. One single calibrated [28] examiner (M.V.) performed the full-mouth measurements using a periodontal probe (UNC-15 Hu-Friedy, Chicago, IL, USA) at six points on every present tooth (mesio-buccal, mid-buccal, disto-buccal, mesio-lingual, mid-lingual and disto-lingual). The average PPD and CAL (in mm) and the overall frequencies (in %) for BOP and PI were calculated for all patients.

Participants were randomly assigned into two groups: 1) LA group, consisting of 19 patients receiving the adjuvant local antibiotics during NSPT; 2) SA group, with 19 patients receiving the adjuvant systemic antibiotics during NSPT. After meeting all the inclusion criteria, prior to treatment, allocation of patients was done by a different investigator (I.M.) who was not involved in examining or treating the patients. This allocation concealment was done in order to eliminate sampling bias where the one investigator (M.V.) examined all patients and another (A.Dj.K.) treated all patients. During the course of the treatment, outcome assessor (M.V.) was blinded to the treatment allocations. The patients were randomly allocated by drawing opaque sealed envelopes with cards coded with LA and SA in order to determine one antibiotic protocol. All NSPTs were performed by a single therapist (A.Dj.K.). Both patients and the therapist were strictly instructed not to inform the examiner if they had received systemic or locally delivered antibiotics. The examiner (M.V.) had no access to dental records and was not present when NSPT or administration of antibiotics in the LA group was performed. The allocation to the intervention group was not revealed until the data set had been locked.

Prior to NSPT, the samples of subgingival gingival crevicular fluid (GCF) were taken from each participant, from the selected periodontal pocket in the premolar/molar region. The pocket with the highest PPD, exceeding 5 mm, always in the first quadrant, was selected for sampling. In the relatively dry working field the paper points No. 30 (DiaDent; Cheongju-si, South Korea) were inserted in periodontal pocket for at least 30 s and transferred into sterile 1.5 ml microcentrifuge tubes (Eppendorf; Eppendorf, Hamburg, Germany) containing RNAlater stabilization solution (RNAlater; Thermo Fisher Scientific, Waltham, MA, USA), refrigerated (2–8 °C) overnight, after which the solution was removed and the samples were stored at −80 °C pending further analysis.

### 2.2. Non-surgical periodontal therapy

After GCF collection, all patients underwent NSPT according to the *full-mouth disinfection* protocol [29]. The adjuvant antibiotic therapy was followed:

- In the LA group, the combination of piperacillin + tazobactam (Gelcide; Italmed MedTechDental, Florence, Italy) was delivered 24 h after the NSPT completion in order to allow relatively dry working field without post-interventional bleeding. The single-delivery protocol for the local antibiotic solution was used. The application of the solution in the periodontal pockets with PPD ≥ 4 mm was performed using a syringe with a blunt needle in six points around the tooth, three buccally and three orally (mesio-buccal, mid-buccal, disto-buccal, mesio-oral, mid-oral, disto-oral). The application continued until liquid flowed over the gingival margin. The local antibiotic delivery was carried out in all regions of both jaws during the same visit; afterwards, additional drying of the area was performed during the next 5 minutes. Patients were instructed not to rinse their mouth with water 15 minutes after the procedure.
- In the SA group, systemic antibiotics were prescribed after NSPT. The combination of amoxicillin (Amoxicillin; Hemofarm, Vršac, Serbia) 500 mg three times daily and metronidazole (Metronidazole; Alkaloid, Skopje, North Macedonia) 400 mg three times daily was used in a 7-day regimen [13]. In order to ensure patients’ compliance in following the systemic antibiotics regimen strictly, all patients from the SA group filled in the daily logbook of timing and pills intake during the course of the treatment.

The patients were scheduled for follow-up appointments six months after the treatment to perform control periodontal charting and GCF sampling.

### 2.3. Laboratory procedures

Both RNA and DNA were extracted from the samples collected from the patients at the baseline and at the six-month follow-up appointment. For both extractions, TRIzol reagent (TRIzol; Thermo Fisher Scientific, Waltham, MA, USA) was used according to the manufacturer’s recommendations.

DNA samples were used for total bacteria quantification by quantitative PCR (qPCR) as described previously [30]. Briefly, amplification of highly conserved target regions of the 16S rRNA gene was performed using a universal SYBR Green Supermix (SsoAdvanced Universal SYBR Green Supermix; Bio-Rad, Hercules, CA, USA) in the presence of the following primers: Fw 5′-TCCTACGGGAGCACAGT′-3 and Rv 5′GGACTACCAGGGTATCTAATCCTGTT-3′. The reference strain used for the standard curve analysis was *Prevotella melaninogenica* (ATCC 25845), and the results were expressed as total gene copy number.

From the RNA samples, cDNA was synthesised using a reverse transcription kit (RevertAid First Strand cDNA Synthesis Kit; Thermo Fisher Scientific, Waltham, MA, USA) and 1 µg of total RNA. Relative gene expression of IL-17 and TNF-α was evaluated using quantitative PCR analysis, as described previously [31], normalised against glyceraldehyde-3-phosphate dehydrogenase (GAPDH) gene expression.

### 2.4. Reproducibility and methodological details

All materials used, including specific brands, models, and dosages, are explicitly detailed in the Methods section. The local antibiotics, piperacillin + tazobactam (Gelcide; Italmed MedTechDental, Florence, Italy), were applied using a syringe with a blunt needle, in six points per tooth, until liquid overflowed over the gingival margin, with application performed in all regions of both jaws during the same session. Systemic antibiotics comprising amoxicillin (500 mg) and metronidazole (400 mg) combination were administered orally three times daily for 7 days, with patient compliance monitored via daily logbooks. Both antibiotics and other reagents, such as TRIzol (Thermo Fisher Scientific), SYBR Green Supermix (Bio-Rad), and reverse transcription kits (RevertAid; Thermo Fisher Scientific), were used following manufacturer instructions. The periodontal probing depth (PPD), clinical attachment level (CAL), bleeding on probing (BOP), and plaque index (PI) were recorded with a standardized periodontal probe (UNC-15 Hu-Friedy), by a calibrated examiner, following standardized measurement points on each tooth. Subgingival plaque sampling involved inserting sterile paper points (No. 30; DiaDent) into the periodontal pocket for at least 30 seconds, then transferring to RNAlater stabilization solution (Thermo Fisher Scientific), and storing samples at −80°C until analysis. Molecular procedures included RNA and DNA extraction per manufacturer instructions, with DNA used for bacterial quantification by qPCR targeting 16S rRNA gene regions (primers: Fw 5′-TCCTACGGGAGCACAGT-3′, Rv 5′-GGACTACCAGGGTATCTAATCCTGTT-3′), and RNA for cytokine expression analysis via reverse transcription and qPCR. All procedures, from sample collection to laboratory analysis, were performed in accordance with standard protocols and manufacturer guidelines, ensuring reproducibility and consistency across all samples.

### 2.5. Statistical analysis

All statistical analyses were performed on an intention-to-treat basis; however, in this study, no dropouts were reported during the observational period. Statistical analyses were performed using statistical software (GraphPad Prism version 9.0; GraphPad Software, San Diego, CA, USA) and (IBM SPSS Statistics version 26.0; IBM, Armonk, NY, USA). For numerical data, mean values, medians, standard deviations (SD) and ranges were used for description. The distributions of all outcome values were examined using the Kolmogorov–Smirnov normality test, and since none followed a normal distribution, measurements at the baseline and the sixth-month follow-up within the two treatment groups were compared using Wilcoxon signed-rank test. Comparisons in measurements between the two treatment groups were done using the Wilcoxon rank-sum test (Mann–Whitney U test). The significance was set at *p* < 0.05.

## 3. Results

### 3.1. Clinical results

**Fig 2:**
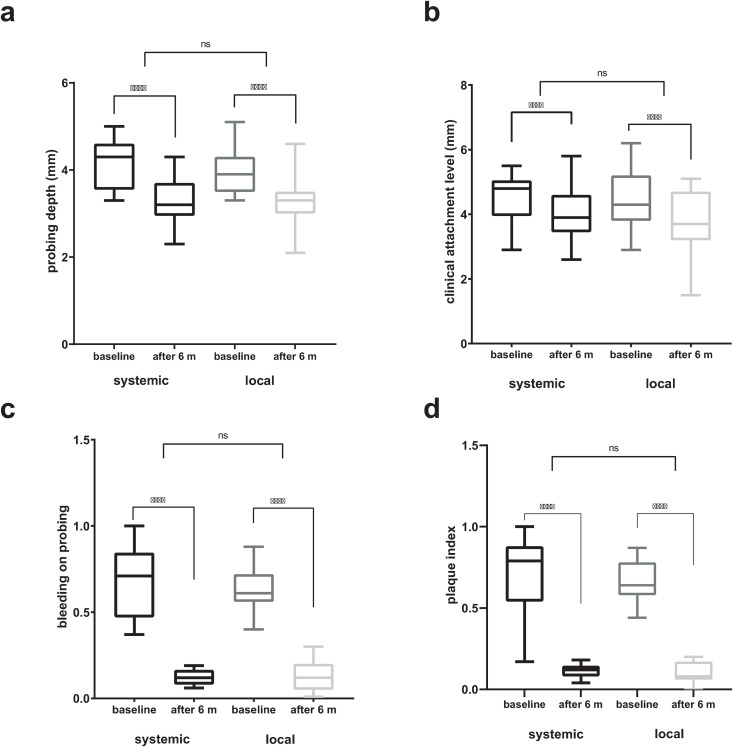
a-d. Difference in (a) probing depth; (b) clinical attachment level; (c) bleeding on probing, and (d) plaque index values between baseline and six months after treatment using systemic and local antibiotic treatment. **** - *p* values less than 0.001; ns – not significant.

The present study included 38 individuals in total, of which 60.5% were female and 39.5% were male. Out of 19 patients allocated into LA group, 11 were female and 8 male, while the SA group consisted of 12 female and 7 male participants. The mean values and standard deviations for clinical measurements for PPD, CAL, BOP and PI were presented in Table 1. No significant difference was observed between groups receiving local or systemic antibiotic treatment at the beginning, nor after six months (*p* > 0.05). However, both treatments led to a highly significant improvement in all clinical measurements after six months (*p* < 0.001 for all comparisons, Fig 2a–d).

**Table 1:** Mean Values and Standard Deviations (Mean [±SD]) of Clinical Parameters: periodontal probing depth (PPD), clinical attachment level (CAL), bleeding on probing (BOP), and plaque index (PI) at baseline and at the 6-month follow-up in local (LA) and systemic (SA) antibiotic group.

Clinical parameter	LA group		SA group	
	Baseline Mean [±SD]	6-month follow-up Mean [±SD]	Baseline Mean [±SD]	6-month follow-up Mean [±SD]
Periodontal Probing Depth (PPD)	3.932 [±0.536]	3.253 [±0.505]	4.084 [±0.583]	3.289 [±0.548]
Clinical Attachment Level (CAL)	4.400 [±0.845]	3.832 [±0.906]	4.500 [±0.766]	4.032 [±0.803]
Bleeding On Probing (BOP)	0.631 [±0.121]	0.123 [±0.084]	0.680 [±0.187]	0.124 [±0.044]
Plaque Index (PI)	0.654 [±0.128]	0.101 [±0.064]	0.704 [±0.253]	0.115 [±0.041]

* LA – local antibiotic; SA – systemic antibiotic.

### 3.2. Total bacterial count

**Fig 3:**
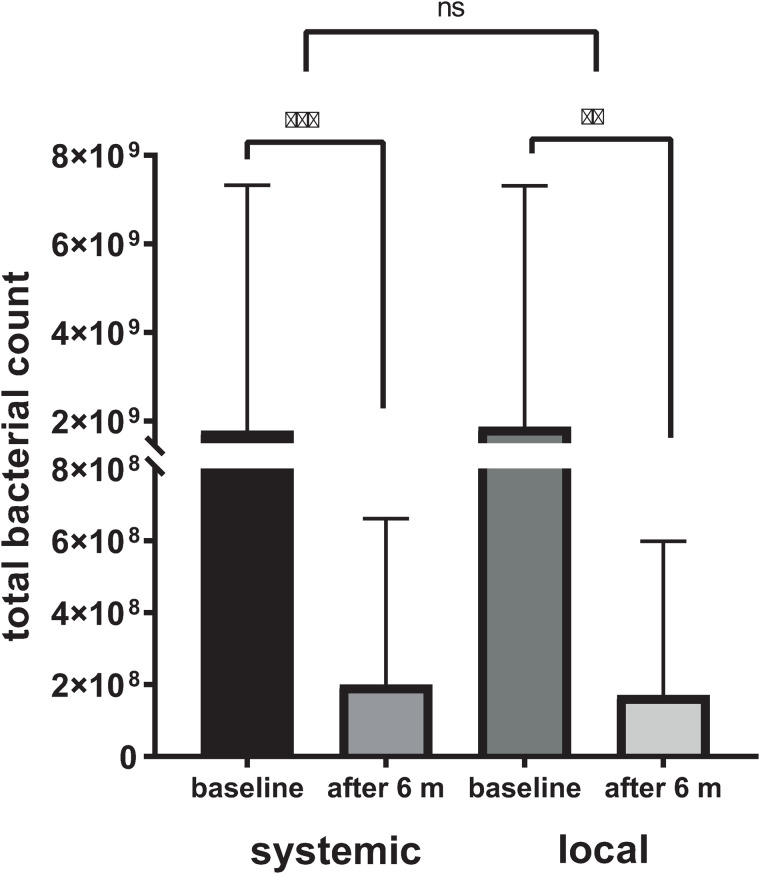
Total bacterial count values at baseline and six months after treatment using systemic and local antibiotic treatment. ** - *p* = 0.013; *** - *p* = 0.001; ns – not significant.

The clinical results were also reflected at the bacterial level, as evidenced by a significant reduction in the total bacterial count. Namely, an approximately ten-fold decrease of total mean [±SD] bacterial count was observed after both local (from 1.87 × 10^9^ [±5.44 × 10^9^] at baseline to 1.71 × 10^8^ [±4.28 × 10^8^], *p* = 0.001) and systemic antibiotics’ treatment (from 2.18 × 10^9^ [±5.39 × 10^9^] at baseline to 2.28 × 10^8^ [±4.47 × 10^8^], *p* = 0.013), however, without significant differences between the two groups after the six-month follow-up (*p* > 0.05) (Fig 3).

### 3.3. Relative gene expression of pro-inflammatory cytokines

**Fig 4:**
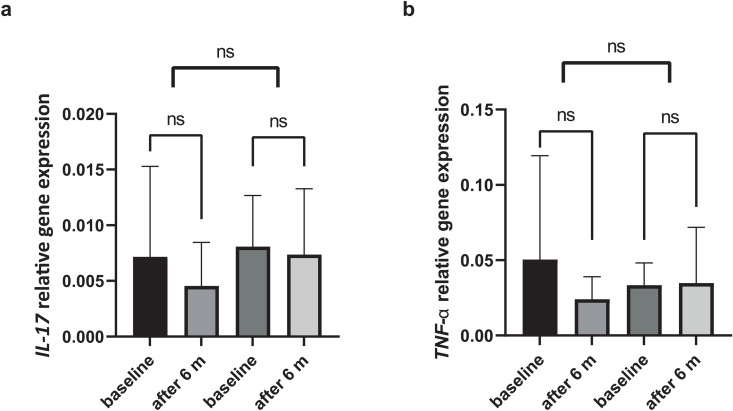
Difference in (a) IL-17, and (b) TNF-α relative gene expression levels between baseline and six months after treatment using systemic and local antibiotic treatment. *ns – not significant.

Although a reduction in local inflammation was expected based on the abovementioned findings, this was not reflected at the gene expression level for selected pro-inflammatory cytokines. To be more precise, the relative gene expression levels of IL-17 and TNF-α did not differ significantly between the two applied antibiotic protocols, nor were they affected significantly by the treatment itself (*p* > 0.05) (Fig 4).

## 4. Discussion

NSPT reduces the infectious burden in periodontal pockets, subsequently breaking the chain of the disease progression. However, despite NSPT alone lowering the number of some periodontal pathogens, it does not seem to alter the composition of the subgingival biofilm sufficiently for the beneficial bacterial species to colonise the periodontal pockets more definitively [16]. Also, all other oral surfaces demand anti-infective treatment as well, since anaerobes were found to reside in aerobic environments of the mouth, such as tongue, saliva and oral mucosa [32]. Hence, for achieving the ecological transition [16], the adjuvant use of antibiotics has been recommended during NSPT [10,16,20]. To the best of our knowledge, the present study was the first to compare the clinical and microbiological effects of the adjunctive use of piperacillin + tazobactam vs. amoxicillin and metronidazole combination in NSPT. This study serves as an educational example of how carefully chosen antibiotic protocols can influence clinical outcomes in periodontal therapy. It highlights the importance of understanding the microbiological rationale behind adjunctive treatments to optimize patient care and systemic health.

The present study demonstrated the significant improvement of all clinical periodontal parameters six months following NSPT, accompanied by both locally and systemically delivered antibiotics. Importantly, no significant differences were observed between the two groups regarding clinical parameters, neither at the beginning of the therapy, nor six months afterwards. Therefore, the null hypothesis was not rejected. In addition, the application of both antibiotic protocols significantly reduced total bacterial count, although no significant differences between the two groups were noted six months later.

Similarly to the present report, the improvement of periodontitis clinical presentation following the adjuvant use of amoxicillin and metronidazole combination in NSPT has been thoroughly scientifically documented. Precisely, the significant advancement of clinical parameters, accompanied by deep sites further reduction from 3 months to even 1-year post-treatment, was demonstrated [16,33,34]. Also, a more recent meta-analysis confirmed the significant reduction of PPD, CAL, BOP, pocket closure and frequency of residual pockets, especially after adjuvant use of amoxicillin and metronidazole combination with NSPT [10]. Moreover, the total bacterial count lowering in our study complied with earlier research that reported the decrease of bacterial complexes harbouring pathogens and an increase of those containing beneficial species during a three-to-12-month period after NSPT with adjuvant amoxicillin and metronidazole combination [16,33].

When considering the effectiveness of the adjuvant local antibiotics with NSPT, the state of the art on this topic remains somewhat controversial. The results of this study were in line with a recent meta-analysis reporting the beneficial effect of locally administered antibiotics during NSPT on clinical outcomes [20]. However, some earlier reports found the use of local antibiotics not to be very favourable, with only limited clinical benefits reported when using adjuvant local antibiotics in comparison to NSPT alone [35]. Exactly, mean improvements of certain clinical parameters were often limited to tenths of millimetres when various local drug delivery systems were used [36,37].

Previous literature reports were assessing a wide variety of local antimicrobial preparations, with different antimicrobials’ delivering systems, rendering their analysis rather complicated. Importantly, unlike many available reports, the combination of piperacillin + tazobactam was used in the present study. Piperacillin is a semisynthetic, extended-spectrum ureidopenicillin whose susceptibility to hydrolysis was reduced by combining it with tazobactam, an irreversible inhibitor of bacterial beta-lactamases [38]. High activity against anaerobes has been reported for the piperacillin + tazobactam combination, allowing its beneficial use for the treatment of polymicrobial anaerobic infections [38,39]. In addition, piperacillin + tazobactam had a better response *in vitro* against periodontopathogenic bacteria compared with that of amoxicillin/clavulanic acid and minocycline [40]. Finally, the substantial reductions in *Treponema denticola*, *Fusobacterium nucleatum spp. Polymorphum*, *Parvimona micra*, and *Fusobacterium periodonticum* has been demonstrated after the use of adjunctive piperacillin + tazobactam compared with the conventional NSPT only [41].

The current literature provides only scarce information on the adjuvant use of locally delivered piperacillin + tazobactam during NSPT. Compared to our observations, another study also showed a significantly lower composite count of selected pathogens during the follow-up [41]. Although, as opposed to the results of the present study, the same trial failed to demonstrate the significant short-term differences in PPD or BOP six months after the therapy [41]. The differences in design between our study and the aforementioned trial should be put forward, as the authors observed only a limited number of deeper pockets per patient, while in our study, a full-mouth disinfection protocol was performed with local antibiotics application in all periodontal pockets ≥ 4 mm. Importantly, even shallow pockets could be highly colonised with several periodontal pathogens; therefore, the need for anti-infective treatment of shallow pockets as well arises to prevent an early bacterial re-colonisation and allow the desired complete ecological shift in the oral environment [16].

It might be stated that the locally delivered antibiotic protocol used in the present study could be beneficial in terms of maintaining the clinical results short-termly and postponing the periodontal pathogens’re-emergence with subsequent periodontal inflammation recurrence. Particularly, the form of locally delivered piperacillin + tazobactam used in this study might be advantageous in terms of longer sustainability (7–10 days) at the place of application [42], while most locally delivered antimicrobials suffer from the inability to maintain substantivity for more than one week [35]. On the other hand, the study design of the present research indicated that a single delivery of local antibiotics led to comparable effects to systemic antibiotics six months after initial therapy, both given adjunctively at the time of NSPT, not later.

Conversely, one of the few studies in the available literature on the use of piperacillin + tazobactam in NSPT performed clinical controls up to six months after the initial periodontal therapy, although NSPT and administration of local antibiotics were repeated at three months. The authors concluded that despite local adjunctive use of piperacillin + tazobactam improving clinical outcomes of NSPT compared to NSPT alone, these results were not maintained over time, and so a more persistent local application was necessary [40]. The results of the present and aforementioned study are not easy to compare and contrast due to their very distinct designs. However, a longer follow-up period would be necessary to elucidate the stability of the therapy effects of locally delivered piperacillin + tazobactam over time.

As for immunological parameters evaluated in this study, no significant differences in IL-17 and TNF-α relative level were reported, neither between the used antibiotic protocols, nor did the applied treatment have an impact on them. Despite the level of pleiotropic pro-inflammatory cytokine TNF-α has been used in identification, severity estimation, and periodontitis progression [5], our results were supported by the previous work, concluding that TNF-α did not appear to be valuable in monitoring the periodontal healing, since no consistent TNF-α reduction was verified after conventional therapy [43]. Interestingly, TNF-α polymorphisms were linked to the increased risk of periodontitis in some population groups [44]. The possibility was proposed that these genetic variations at least partly explain why the improvement in periodontal clinical records was not associated with decreased TNF-α production [43].

Furthermore, TNF-α is secreted also by the Th-17 cells, along with their signature cytokine IL-17 and numerous other pro-inflammatory mediators [45]. The participation of IL-17, together with TNF-α, in the pathogenesis of bone loss in periodontitis has already been suggested [7,31,46]. However, similar to the results of our study, IL-17 levels were also reported not to be significantly influenced by NSPT [47,48]. Considering their mutual roles in the periodontitis pathogenesis and somewhat shared source of origin, it might be cautiously concluded that levels of TNF-α and IL-17 could be band together in responding to periodontal therapy, possibly without following the improvement of clinical presentation of the disease itself. Since the present study is limited by the number of participants and the observational period, this hypothesis needs to be further explored.

It should be highlighted once more that the present study failed to demonstrate any significant differences between the two antibiotic protocols on clinical, microbiological and cytokine presentation six months after NSPT. Recently, a retrospective study demonstrated similar clinical and microbiological observations three months following NSPT with either systemically or locally delivered antibiotics [23]. The available literature was mostly focused on the limited usefulness of local antibiotics, on cases of localised, remaining deep pockets, and on supportive periodontal therapy. The uncertain cost-benefit ratio and inability to reach all infected tissues in the mouth participating in the ecological shift have been particularly stressed as an important drawback of local antibiotics [16,49]. At this point it should be also bared in mind that various treatment methods during NSPT (hand instruments, Er:YAG-laser, sonic, and ultrasonic scalers) resulted in a comparable reduction of the evaluated periodontal pathogens, without any antimicrobial regimens [50].

On the other hand, in spite of the above-mentioned well-documented benefits of adjuvant systemic antibiotics, the significant adverse effects, increased bacterial resistance, and uncertain patient compliance have been emphasised, suggesting careful considerations prior to their use [16,49,51]. Given that both local and systemic antibiotics used in the present study led to comparable clinical and microbiological improvement, the local application of piperacillin + tazobactam seems to be a promising alternative to the conventional systemic amoxicillin and metronidazol protocol. Additionally, antibiotic resistance to piperacillin + tazobactam appeared to be reduced compared to amoxicillin/clavulanic acid [52]. It might be stated that the strength of the present study is that it was the first clinical research that intended to compare this particular combination of adjunctive locally delivered antibiotics and conventional systemic antibiotics during NSPT, with similar clinical benefits. This would surely be in line with the need for the reduction of systemic antibiotics use globally and restraining the rise of bacterial resistance.

Even though the observations made in this study appear encouraging, further investigation on a larger cohort with a longer follow-up period is very much needed to bring a more conclusive statement. The present study is limited not only by its sample size and follow-up time, but also by the study design itself. The protocol of either prescribing systemic antibiotics or delivering them locally interfered with the possibility of having a double-blinded trial. In addition, the present study was also limited to evaluating just a total bacterial count, along with only two pro-inflammatory cytokines. Targeting specific bacterial species and measuring the relative gene expression levels, with protein levels as well, of more numerous pro-inflammatory and anti-inflammatory mediators would be very much needed for further confirmation or contradiction of the results of this research.

## 5. Conclusions

Within the limitations of the present study, the adjuvant use of piperacillin + tazobactam during NSPT led to a similar reduction of PPD to the conventional use of amoxicillin and metroniazol combination six months following the completion of the initial periodontal therapy. Furthermore, other clinical parameters likewise demonstrated the comparable improvement, coupled with a lowering of the total bacterial number and relative levels of investigated cytokines.

## Supporting information

S1 File CONSORT checklist. (DOC)
